# Ethical Criteria for the Admission and Management of Patients in the ICU Under Conditions of Limited Medical Resources: A Shared International Proposal in View of the COVID-19 Pandemic

**DOI:** 10.3389/fpubh.2020.00284

**Published:** 2020-06-16

**Authors:** Vittoradolfo Tambone, Donald Boudreau, Massimo Ciccozzi, Karen Sanders, Laura Leondina Campanozzi, Jane Wathuta, Luciano Violante, Roberto Cauda, Carlo Petrini, Antonio Abbate, Rossana Alloni, Josepmaria Argemi, Josep Argemí Renom, Anna De Benedictis, France Galerneau, Emilio García-Sánchez, Giampaolo Ghilardi, Janet Palmer Hafler, Magdalena Linden, Alfredo Marcos, Andrea Onetti Muda, Marco Pandolfi, Thierry Pelaccia, Mario Picozzi, Ruben Oscar Revello, Giovanna Ricci, Robert Rohrbaugh, Patrizio Rossi, Ascanio Sirignano, Antonio Gioacchino Spagnolo, Trevor Stammers, Lourdes Velázquez, Evandro Agazzi, Mark Mercurio

**Affiliations:** ^1^Institute of Philosophy of Scientific and Technological Practice (FAST), Campus Bio-Medico University of Rome, Rome, Italy; ^2^Department of Medicine, Institute of Health Sciences Education, McGill University, Montreal, QC, Canada; ^3^Research Unit of Medical Statistic and Molecular Epidemiology, Campus Bio-Medico University of Rome, Rome, Italy; ^4^Department of Business, Law and Society, St Mary's University, London, United Kingdom; ^5^Institute for Family Studies & Ethics, Strathmore University, Nairobi, Kenya; ^6^Fondazione Leonardo, Civiltà delle Macchine, Rome, Italy; ^7^Section of Infection Diseases, Department of Healthcare Surveillance and Bioethics, Catholic University of Sacred Heart, Rome, Italy; ^8^Bioethics Unit, Italian National Institute of Health, Rome, Italy; ^9^Department of Internal Medicine, Virginia Commonwealth University School of Medicine, Richmond, VA, United States; ^10^Hospital Clinical Direction, Campus Bio-Medico University of Rome, Rome, Italy; ^11^Division of Medicine, Gastroenterology and Hepatology Department, University of Pittsburgh, Pittsburgh, PA, United States; ^12^Department of Medicine, Universitat Internacional de Catalunya, Barcelona, Spain; ^13^Department of Obstetrics, Gynecology and Reproductive Sciences, Yale University School of Medicine, New Haven, CT, United States; ^14^Department of Political Sciences, Ethics and Sociology, University CEU Cardenal Herrera, Valencia, Spain; ^15^Teaching and Learning Center, Yale University School of Medicine, New Haven, CT, United States; ^16^Department of Medicine, Solna, Karolinska Institutet, Center for Molecular Medicine, Stockholm, Sweden; ^17^Department of Philosophy, Universidad de Valladolid, Valladolid, Spain; ^18^Department of Laboratories, Bambino Gesù Children's Hospital, IRCCS, Rome, Italy; ^19^Prehospital Emergency Medical Service (SAMU 67), Strasbourg University Hospital, Strasbourg, France; ^20^Center for Clinical Ethics, Insubria University, Varese, Italy; ^21^Instituto de Bioética de la Facultad de Ciencias Médica, Pontificia Universidad Católica Argentina, Buenos Aires, Argentina; ^22^School of Law, University of Camerino, Macerata, Italy; ^23^Department of Psychiatry, Yale University School of Medicine, New Haven, CT, United States; ^24^Central Medical Department, National Institute for Insurance against Accidents at Work (INAIL), Rome, Italy; ^25^School of Medicine, Catholic University of Sacred Heart, Rome, Italy; ^26^Centre for Bioethics and Emerging Technologies, Institute of Theology, St Mary's University, London, United Kingdom; ^27^Interdisciplinary Bioethics Center, Universidad Panamericana, Mexico City, Mexico; ^28^Program for Biomedical Ethics, Yale University School of Medicine, New Haven, CT, United States

**Keywords:** COVID-19, Intensive Care Unit, disaster medicine, ethical triage criteria, common good

## Introduction

The present pandemic has exposed us to unprecedented challenges that need to be addressed not just for the current state, but also for possible future similar occurrences. It is worth pointing out that discussions on the allocation of medical resources may not necessarily refer to an exception, but, unfortunately, to a regular condition for a large part of humanity (1). The criteria for admission to an Intensive Care Unit (ICU) setting generally take into account multiple factors. There must be a diagnostic and prognostic basis for the decisions made, considering both biological factors and patient values and wishes. Furthermore, the decision-making process should, whenever possible, respect the patient's advance directives as well as the relationship with the patient's family or attorney. Therapeutic neglect should be avoided.

Having applied standard clinical evaluation criteria for the appropriate treatment of patients with COVID-19, including consideration of prognosis, if a hospital then finds itself unable to provide optimal treatment (e.g., due to a disproportion between the number of patients and the availability of beds, healthcare providers, ventilators, and drugs in the ICU), it becomes necessary to evaluate, case by case, how to achieve justice and the best possible good for the greatest number of patients. It is therefore mandatory to explore alternative solutions; these include increasing available beds and healthcare providers, implementing alternative, though suboptimal, approaches (where appropriate), transferring patients to other clinical units, etc. Making these decisions properly also involves the recovery of the political role of medicine and science (2).

If the imbalance between needs and resources reaches a critical level, an emergency triage protocol, following the operational and ethical indications of “disaster medicine,” should be activated. These have been deployed in major and serious natural (earthquakes or tsunamis for example) and technological (factory explosions, public transport accidents for example) disasters, as well as following terrorist attacks (3, 4). The question of the feasibility of developing a clinical evaluation algorithm to support the decision-making of the triage team remains open, though many such protocols have been written.

According to the above, we propose the following five ethical criteria for the triage of patients in conditions of limited resources, such as the COVID pandemic. They are the result of an interdisciplinary and intercultural dialogue between specialists from different disciplines. Several of the authors are working in the main epicenters of the crisis and currently are playing a central role in the bioethical, clinical, social and legal aspects of the management of the COVID-19 pandemic.

## Ethical Triage Criteria

- We take the following three general principles as evaluative references: (a) the good of a single patient should be considered in the framework of the common good. Common good means the good of all people and of the whole person. It is rooted in the idea of human dignity, which gives birth to the humanitarian imperative conveyed in the first core principle of “disaster medicine”; the common good also means that, in a Global Health framework, patients are not just isolated individuals but persons with strong ties to their communities, and therefore both patient and community need to be taken into account (5); (b) no one must be abandoned or discriminated against for any reason (6); (c) before denying a necessary referral of a patient to an ICU, due to lack of resources, it is required to consider alternatives both for the immediate case and, based on the experience gained, for similar future cases.
- Appropriate assistance to any person in need of medical care should be provided whenever possible. In critical situations, the criteria for determining priority are the urgency and severity of the clinical situation. Consideration should also be given to the effectiveness and proportionality of the medical intervention, with the goal of obtaining the greatest possible benefit for the greatest number of patients.
- Triage must be carried out on a case-by-case basis, with reference not only to the patient's clinical condition but also to the availability of resources in the hospital. Possible transfer initiatives to other larger and better resourced national or foreign intensive care units must also be considered. Triage must not proceed using a standardized approach where the sole decision-making criteria is age (7).
- Inappropriate treatments are not acceptable.
- Adequate forms of palliative and spiritual care must be assured, where necessary.

## Author Contributions

The manuscript is an original work of all authors. All authors made a significant contribution to this paper and have read and approved the final version of the manuscript.

## Conflict of Interest

The authors declare that the research was conducted in the absence of any commercial or financial relationships that could be construed as a potential conflict of interest. The handling editor declared a shared affiliation, though no other collaboration, with one of the authors JA.
